# Achieving Congruence among Reference Laboratories for Absolute Abundance Measurement of Analytes for Rare Diseases: Psychosine for Diagnosis and Prognosis of Krabbe Disease

**DOI:** 10.3390/ijns6020029

**Published:** 2020-03-31

**Authors:** Zackary Herbst, Coleman T. Turgeon, Chad Biski, Hamid Khaledi, Nancy B. Shoemaker, Patrick D. DeArmond, Sara Smith, Joseph Orsini, Dietrich Matern, Michael H. Gelb

**Affiliations:** 1 Departments of Chemistry and Biochemistry, University of Washington, Seattle, WA 98195, USA; zherbst@uw.edu (Z.H.); hamidkh@uw.edu (H.K.); 2 Biochemical Genetics Laboratory, Mayo Clinic, Rochester, MN 55905, USA; turgeon.coleman@mayo.edu (C.T.T.); matern@mayo.edu (D.M.); 3 Newborn Screening Program, Wadsworth Center, New York State Department of Health, Albany, NY 12201-2002, USA; chad.biski@health.ny.gov (C.B.); joseph.orsini@health.ny.gov (J.O.); 4 GelbChem LLC, 4000 Mason Road, Seattle, WA 98195, USA; 5 Department of Pathology and Laboratory Medicine, Nationwide Children’s Hospital, Columbus, OH 43205, USA; nancy.shoemaker@nationwidechildrens.org (N.B.S.); patrick.dearmond@nationwidechildrens.org (P.D.D.); 6 PerkinElmer Genomics, Pittsburgh, PA 15275, USA; Sara.Smith@PERKINELMER.COM

**Keywords:** newborn screening, biomarkers, mass spectrometry, Krabbe disease

## Abstract

Measurement of the absolute concentration of the biomarker psychosine in dried blood spots (DBS) is useful for diagnosis and prognosis of Krabbe disease and to support newborn screening of this leukodystrophy. As for assays for more common diseases, it is important to achieve congruence when multiple clinical laboratories provide testing. Four clinical laboratories, one research laboratory, and a commercial vendor collaborated with the goal to achieve congruence in quantitative psychosine measurement in DBS. A set of DBS calibrators was prepared by a single vendor and used in each reference laboratory to make a standard curve of measured psychosine in DBS versus the stated calibrator psychosine level. Congruence between the participating five laboratories was achieved using the psychosine DBS calibrators. This allowed application of disease-specific reference ranges obtained by the reference laboratory with the most extensive data by the other reference laboratories. Congruence between multiple reference laboratories in the measurement of the absolute concentration of biomarkers in DBS (and by extension other samples) is possible by the use of a common set of DBS calibrators.

## 1. Introduction 

**Figure 1:**
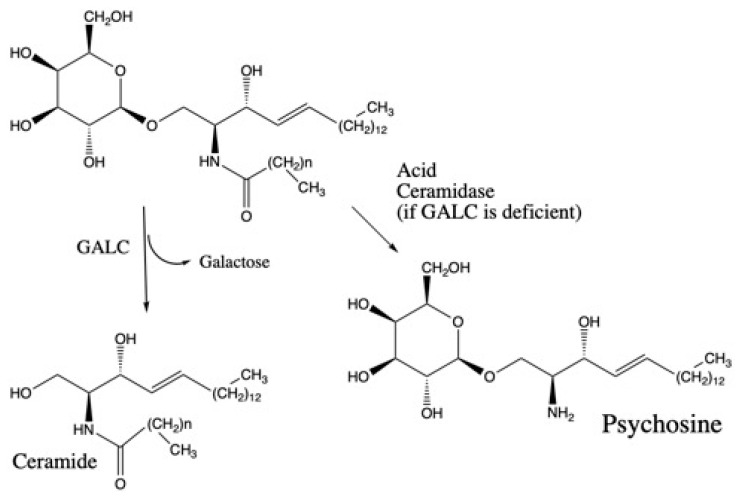
Galactocerebrosidase (GALC)-catalyzed reaction and direct conversion of galactosylceramide to psychosine when GALC is deficient as proposed [4].

The glycosylsphingolipid psychosine has emerged as a biomarker for diagnosis and prognosis of Krabbe disease, a rare lysosomal storage disorder caused by deficiency of galactocerebrosidase (GALC) [1,2,3]. GALC removes galactose from galactosylceramide as part of the lysosomal re-cycling of glycosphingolipids that are rich in myelin (Figure 1). The next step in the breakdown pathway is release of the fatty acyl group from ceramide by acid ceramidase (Figure 1). Recent studies have suggested that when GALC is deficient, acid ceramidase removes the fatty acyl chain from galactosylceramide to generate psychosine (Figure 1) [4]. Psychosine is thought to be a key cytotoxic agent that is responsible for loss of oligodendrocytes and Schwann cells that is the hallmark of Krabbe disease [4].

Newborn screening for Krabbe disease is done by first-tier measurement of GALC enzymatic activity in dried blood spots (DBS). Because the majority of cases with low enzymatic activity samples have pseudodeficient GALC activity due to common pseudodeficiency *GALC* gene variants [5], reliance on low GALC activity alone is not sufficient for newborn screening. Psychosine has been shown to be a valuable biomarker when measured in DBS to detect newborns with infantile Krabbe disease [1,2,3], and measuring psychosine as a second-tier test in newborn screening can clarify the vast majority of these low-GALC activity cases as false positives [6]. A few newborn screening laboratories include psychosine analysis as a second-tier method [6], but most programs refer patients to diagnostic centers either when GALC activity is found to be reduced or when molecular genetic analysis of the *GALC* gene is used as a second tier test and reveals a genotype with known pathogenic mutations or variants of uncertain significance. Psychosine may then be measured during follow-up of the abnormal newborn screening result. 

Psychosine has also been evaluated as a biomarker to predict the severity of Krabbe disease [1,3,7,8,9]. For patients tested at the Mayo Clinic, those with psychosine of greater than ~10 nM in DBS are considered at high risk to develop early-onset Krabbe disease (symptoms during the first year of life), whereas patients who develop later-onset Krabbe disease typically have psychosine values in the 2–10 nM range. Patients with psychosine < 2 nM are at very low risk to develop Krabbe disease during their lifetime [8].

Psychosine measurement cannot yet replace GALC activity as a primary newborn screening test because it requires a mass spectrometer with a limit of detection lower than those typically used in newborn screening laboratories. It also requires liquid chromatography coupled to mass spectrometry (LC-MS/MS) with a run time of several minutes per sample, which is not conducive to the necessary high-throughput requirement of newborn screening.

Given the reference ranges for psychosine that have been established using DBS from confirmed patients with Krabbe disease with early and late onset symptoms [8], it is critical for reference laboratories to achieve precise measurement of absolute levels of psychosine in DBS. This is especially the case for Krabbe disease, because of the limited availability of DBS from actual patients with Krabbe disease. Thus, it would be very time consuming for each laboratory to develop its own disease-specific ranges.

Measurement of the amount of a substance by tandem mass spectrometry (MS/MS), either by itself or interfaced with liquid chromatography (LC-MS/MS), is typically done by addition of a known quantity of internal standard to the sample and measurement of the MS/MS signal of the analyte of interest and that of the internal standard. Multiple reaction monitoring (MRM) is usually used to measure the MS/MS signals. The best internal standards are those that are chemically identical to the analyte but differentiated by use of heavy isotopes. These internal standards elute from the chromatography column together with the analyte and thus suffer the same degree of ionization suppression from the additional components in the sample (matrix effect). The use of these internal standards also accounts for losses of analyte during sample processing.

A laboratory can establish reference ranges for an analyte by using a single stock of internal standard. Patient samples analyzed following the same protocol and using the same reagents and equipment can be reliably compared to the reference range. This is valid even if the amount of internal standard added to each sample is inaccurate (i.e., equal to the true amount times some constant). This can be seen in the following way. The true amount of analyte in the sample is given by Equations (1) and (2).

(1) $$\;A_{true}=\left(\frac{MRM_{A}}{MRM_{IS}}\right)\;\left(\frac{1}{RRF}\right)\left(IS_{app}\right)\left(\frac{IS_{true}}{IS_{app}}\right)$$

(2) RRF=(MRMAMRMIS)(AtrueIStrue)

here, *A_true_* and *IS_true_* are the true moles of analyte and internal standard, respectively, in the sample, and *MRM_A_* and *MRM_IS_* are the measured MS/MS signals in the analyte and internal standard MRM channels, respectively. *IS_app_* is the apparent moles of internal standard added to the sample, i.e., the moles stated by the investigator and not necessarily equal to *IS_true_* (see below). *RRF,* often called the MS/MS relative response factor, accounts for the fact that the ratio of measured MRM signals for analyte and internal standard may not be equal to the ratio of true moles of analyte and internal standard in the sample because of many possible factors, including instrument tuning parameter variations [10]. The apparent moles of analyte in the sample are given by Equation (3).

(3) $$A_{app}=\left(\frac{MRM_{A}}{MRM_{IS}}\right)IS_{app}$$

*A_app_* is proportional to *A_true_.* The reference laboratory does not need to measure *RRF* and $\frac{IS_{true}}{IS_{app}}$, and can thus obtain values of *A_app_* from patient samples and compare them to the reference range as long as the same batch of internal standard is used.

If a new batch of internal standard is used, its relationship to the original batch needs to be measured. All of this assumes that *RRF* does not change over time, and the degree of *RRF* drift is typically not well known for most MS/MS instruments. MS/MS manufacturers recommend that *RRF* values be measured on a regular basis.

Laboratories could determine their own reference ranges and test sample values based on their *A_app_* values. This would be valid even if values of *RRF* and $\frac{IS_{true}}{IS_{app}}$ differ among the laboratories (which is likely to be the case). However, this has not been practical in the case of rare diseases such as Krabbe disease where the numbers of samples such as DBS from affected patients are very limited. The problem is compounded by the need to examine DBS from newborns (only available from a small subset of newborn screening laboratory that store them), which is what is needed to support newborn screening. For example, one of the reference laboratories in our group has obtained DBS from more than 10 patients with early-onset Krabbe disease and more than 10 DBS from patients with late-onset disease, a second laboratory has obtained less than 6 DBS from patients, all with early-onset disease, and the other laboratories have even fewer samples. The latter laboratories are not in a position to report out possible late-onset Krabbe disease. 

The factor by which $\frac{IS_{true}}{IS_{app}}$ differs from unity could be minimized if all reference laboratories obtained deuterated psychosine IS from the same source in which the true moles of psychosine in the supplied vial is known. As discussed in detail previously, manufacturers often state that a compound is pure, based on analyses that do not guarantee purity by weight [10]. For example, chromatography with UV detection may show that psychosine is the major lipid species in the vial but does not report on impurities that are invisible using this approach (i.e., solvents such as water, trace amounts of solid supports used for chromatographic purification including silica gel, etc.). This is particularly true for internal standards such as deuterated psychosine, where the number of interested laboratories is relatively small and there is little incentive for manufacturers to provide certified reference standards. As described previously, quantitative proton nuclear magnetic resonance (qNMR) is a way to determine the true moles of material in a vial since it allows the protons in a molecule to be counted, relative to those in an available certified internal standard in which the internal standard is well established to be pure by weight [10]. qNMR-certified deuterated psychosine is now available (see Section 2). However, even if all laboratories used qNMR-certified deuterated psychosine as internal standard, there is still the need to establish *RRF* values on each MS/MS instrument, which requires the additional reagent of certified non-deuterated psychosine so that $\frac{A_{true}}{IS_{true}}$ is known and *RRF* can be measured (Equation (2)). There could be additional factors such as pipetting precision, adherence of analytes such as hydrophobic psychosine to container walls, etc.

In this study we report a collaborative effort of four clinical laboratories and one research laboratory to develop a psychosine measurement protocol that ensures congruence between the participating laboratories. As we illustrate this for Krabbe disease, it may serve as an example for other rare diseases where absolute abundances of diagnostic and prognostic biomarkers are measured and samples from patients are very limited.

## 2. Materials and Methods

### Preparation of Psychosine/DBS Calibrators

Venous blood (~100 mL) was collected from a single healthy adult into heparanized tubes, and the hematocrit was determined in the usual way by centrifugation of a 5 mL aliquot. The hematocrit was found to be 50% and was not adjusted. Certified stock solutions (qNMR) of d_0_- and d_5_-psychosine were obtained from Avanti Polar Lipids (Alabaster, AL, ce, Alabaster, AL, USA). Stock solutions of d_0_-psychosine were serially diluted into reagent grade methanol using glass syringes (Hamilton, Reno, NV, USA), such that each syringe was filled by more than 30% of its capacity to minimize errors. Final stock solutions of 0.1, 0.2, 0.5, 1.0, 2.5, 5.0 and 10.0 μM analyte were prepared fresh before use.

**Figure 2:**
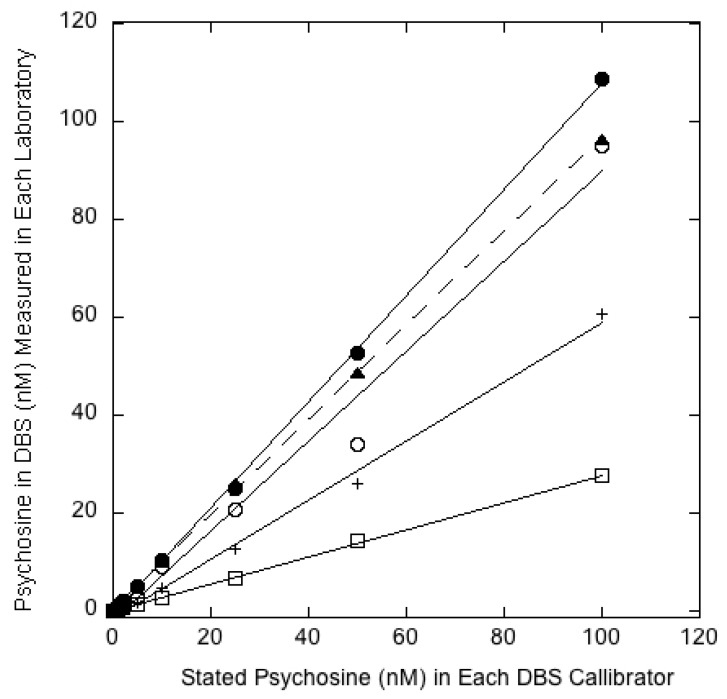
The *y*-axis gives the psychosine value (nM) measured in each laboratory by their LC-MS/MS method. The *x*-axis gives the vendor stated psychosine values (nM) for each dried blood spot (DBS) calibrator: Filled circles/solid line (Nationwide), open circles/solid line (Mayo), filled triangles/dotted line (PerkinElmer), open squares/solid line (NY Wadsworth Center), plus signs/solid line (GelbChem LLC).

Aliquots of heparinized whole blood (11.88 mL delivered with a 1000 μL pipette, were placed into glass vials, each equipped with a magnetic stir bar. With vigorous and continuous stirring, 0.12 mL of methanol stock solution of d_0_-psychosine was added to give the desired target psychosine concentration (0, 1, 2, 5, 10, 25, 50, and 100 nM). The methanol was added over ~30 sec with a Hamilton glass syringe, with the needle tip inserted into the blood. Using a 0.5 mL repeater pipet, 0.5 mL of blood was taken up, and 0.05 mL aliquots were delivered to each filter paper card (Whatmann 10534612 903 Protein Saver Cards from Millipore Sigma (St. Louis, MO, USA) to make 8 blood spots (the first and last 0.05 mL aliquot was delivered to the vial of blood rather than to the filter paper). The process was repeated until most of the blood was spotted. The cards were allowed to dry at room temperature for 2 h and then stored at −20 °C in glass jars with calcium sulfate desiccant. d_0_-Psychosine was measured in 3 mm punches from the DBS calibrators by LC-MS/MS, as described using qNMR-certified d_5_-psychosine as internal standard [2], to provide the standard curve in Figure 2 from the vendor.

## 3. Results and Discussion

After much deliberation, it was decided to take the following approach to best ensure congruence between psychosine values across all participating laboratories. Human blood from a single donor was spiked with known amounts of non-deuterated psychosine from a stock solution made from qNMR-certified material. Spiked psychosine values of 0, 1, 2, 5, 10, 25, 50, and 100 nM were used in order to cover the range of psychosine values measured in control and patient DBS. DBS calibrators were created by spotting spiked blood onto standard 903-grade filter paper. These DBS calibrators were made by a single vendor (see Section 2) and supplied to the participating laboratories for use as calibrators. Each laboratory measured the amount of psychosine in each calibrator DBS using the LC-MS/MS method and stock solution of deuterated psychosine (as internal standard) of their choice. Each laboratory then made a standard curve with measured psychosine on the *y*-axis and quality control psychosine (as stated by the manufacturer) on the *x*-axis. The set of these standard curves from the four clinical laboratories, the research laboratory as well as from the vendor are shown together in Figure 2. It can be seen that the slopes of these standard curves are different in each laboratory, which underscores the lack of congruence noted above. However, this is presumably of no concern since each laboratory pledges to use its own standard curve to determine the psychosine concentrations in patient samples. In this way, even though each of the four laboratories will obtain different assay responses on an identical test sample, they will all report the same psychosine values based on their own standard curve made from the same calibrators. Furthermore, each laboratory includes at least a subset of these DBS calibrators each time they measure psychosine in a set of new patient samples. In this way, any drifts in the analysis, for example a change in *RRF*, will be accounted for.

This approach requires that the true amount of psychosine in the calibrators does not change during storage of the calibrators. In a previous study, it was found that psychosine was stable in freezer-stored DBS for up to 6 months [3], and thus it was decided that an expiration date of 3 months be placed on the calibrators until the extended stability has been determined. It was also decided that when the manufacturer makes a new lot of calibrators, that they be checked side-by-side with the previous lot. Any differences will be assumed to be due to different absolute amounts of psychosine added to the new lot versus the old lot (this makes the reasonable assumption that *RRF* does not change between samples run on the same day). The new lot will be calibrated to the old lot. For example, if the newly made putative 1 nM psychosine DBS standard reads 10% higher by LC-MS/MS than the 1 nM standard in the original lot tested at the same time, the new lot will be specified as a 1.1 nM calibrator. The manufacturer also agreed that when making a new lot of calibrators, both deuterated and non-deuterated psychosine standards that have been certified by qNMR will be used. This will presumably lead to a minimal change in the measured calibrator level of the new lot compared to that of the original lot, and thus, the updated calibrator levels will be very close to those of the original lot. This approach does not guarantee against a slow and systematic drift in calibrator levels over a long period of time, and going forward, it will be important to monitor the lot-to-lot variation over several lots. Hopefully, any changes will be slight and not systematically in one direction.

By using this common set of psychosine DBS calibrators, it is possible for the laboratories with limited access to patient specimens to adopt the reference ranges from the laboratory with the most extensively studied reference ranges [8]. As noted above, this is essential in the case of Krabbe disease where the number of specimens from affected patients is very limited, especially when considering different degrees of disease severity.
